# Supplementary material for: Ancestral role of Pax6 in chordate brain regionalization
Source: Front Cell Dev Biol. 2024 Jul 25;12:1431337. doi: 10.3389/fcell.2024.1431337 (PMC11306081; doi:10.3389/fcell.2024.1431337)
Supplement: Supplementary file 1 [file DataSheet1.PDF]

Supplementary Table 1

zk1770A	TAGGAGCGGCGTGAACCAGCTC
zk1770B	AAACGAGCTGGTTCACGCCGCT
zk2059	(20xA)ATAGCGGCGTGAACCAGCTCG
zk1989QL2	CATAGCGGCGTGAACGGGG
zk614	TTCCCAGAATCTTGGACACGCA
zk2027A	GCAGGGCATAGCGGCGTGAACGGGGGCGTGTTCGTGA
zk2027B	TCACGAACACGCCCCCGTTCACGCCGCTATGCCCTGC
zk1361A	AGTGAGGATCCAAGCAGTGCCAGAACGCCGCC
zk1361B	GCGTCAAGCTTTGCTTATCTGAAGCCGACGTT
zk1979C	TTCTGATCTTCCCTGCCATCA
zk1979D	TGGTCTTTGTTGATGGGGTTG
